# Clozapine for Quetiapine-Refractory Psychosis in Parkinson's Disease: A Long-Term Single-Center Retrospective Study

**DOI:** 10.1155/padi/1068722

**Published:** 2025-03-10

**Authors:** Walter Pirker

**Affiliations:** Department of Neurology, Klinik Ottakring, Vienna, Austria

**Keywords:** clozapine, delusions, hallucinations, Parkinson's disease, psychosis, quetiapine

## Abstract

**Background:** Hallucinations and delusions are severe long-term complications of Parkinson's disease (PD). Clozapine is the only antipsychotic with proven efficacy in PD psychosis (PDPsy) available outside the United States but apprehensions about its adverse event profile result in a substantial underuse of clozapine.

**Objectives:** To investigate the practical use and long-term efficacy of clozapine in severe psychotic disorders in PD.

**Methods:** In this retrospective study, the author used data collected over a period of 20 years and included all PD patients under his care, who were treated with clozapine for psychotic disorders.

**Results:** During the observation period, 41 PD patients (38 with PDPsy, 2 with psychotic depression, and one with schizoaffective disorder) were started on clozapine. They had responded poorly or only transiently to quetiapine. An overnight switch to clozapine was tolerated in most. Maximum clozapine doses ranged from 12.5 to 150 mg (72.9 ± 29.9 mg). A significant reduction in psychotic symptoms was achieved in 2 days to 6 months. Among the cases tolerating clozapine, 10 had a full, 25 had a good, 3 had a moderate, and 2 had a poor clinical response to clozapine. Treatment lasted up to 12 years. The long-term response was full or good in 23, moderate in 3, and poor in 2 patients.

**Conclusions:** Clozapine is often effective in the treatment of psychotic disorders in PD including PDPsy poorly or only transiently responding to quetiapine. Side effects including agranulocytosis are manageable in the majority of cases. Clozapine treatment should not be delayed if other measures against PDPsy prove ineffective.

## 1. Introduction

Hallucinations and delusions represent severe long-term complications of Parkinson's disease (PD), often leading to familial discord and nursing home admission [1, 2]. The development of PD psychosis (PDPsy) may follow a stepwise progression, initiated by vivid dreaming; a false sense of presence and illusions, succeeded by (mostly visual) hallucinations, first with retained insight, later with the loss of insight into their pathological nature; and finally delusions [1, 2]. Mechanisms leading to PDPsy include a complex interplay of neurodegeneration and drug treatment for motor symptoms. Acute PDPsy is often triggered by exogenous factors such as dehydration, infection, or trauma. Endogenous factors include age, duration of PD, and cognitive decline [3].

Crucial steps in the management of PDPsy include a careful search for exogenous triggers, their treatment, reduction or withdrawal of adjuvant PD medication and, depending on the persistence or severity of PDPsy, the introduction of antipsychotics [3]. According to an evidence-based MDS review, only two drugs, clozapine and pimavanserin, show clear-cut efficacy in PDPsy [4], but pimavanserin is not available outside the United States so far [5]. Although the majority of randomized studies using quetiapine in PDPsy were negative, quetiapine was labeled as possibly useful in the MDS review [4] and is still widely used in this indication in Europe. Other antipsychotics including risperidone, olanzapine, and aripiprazole led to substantial worsening of motor symptoms and should be avoided in PD [6]. The rate of extrapyramidal side effects in randomized controlled studies of brexpiprazole in Alzheimer's disease was low but still above placebo [7]. Marked worsening of parkinsonism was observed in a single case following treatment with brexpiprazole [8].

The efficacy of clozapine in PDPsy was demonstrated in two randomized, double-blind, placebo-controlled studies [9, 10] with open long-term follow-up [11, 12]. In the randomized studies, low doses of clozapine (6.25–50 mg daily) led to an improvement of psychosis without relevant worsening of motor symptoms [9, 10]. The French study, which investigated the time-efficacy relationship in detail, found an improvement in psychosis already at 1 week after treatment initiation. Further improvement was seen in the next 3 months and a relapse of psychosis in the majority of patients after clozapine withdrawal [12]. To avoid the risk of placebo treatment these studies excluded patients with very severe PDPsy. In clinical practice, clozapine is the only useful treatment option for patients with severe persecutory delusions and psychotic PD patients with violent behavior. These patients may benefit from higher clozapine doses than the ones used in randomized studies [13].

The risk of agranulocytosis is the most important limiting factor for the use of clozapine. Other adverse events include sedation, drooling, orthostatic hypotension, weight gain ,and an increased risk of venous thromboembolism. Concerns regarding its adverse event profile and challenges associated with the required regular blood monitoring result in a substantial underuse of clozapine [5, 14].

In this retrospective study, the author collected data from all PD patients under his care, treated with clozapine for psychotic disorders over a period of 20 years. The aims of the study were to investigate the practical use, long-term efficacy, and adverse effect profile of clozapine in psychotic disorders in PD. This included not only patients with PDPsy but also PD patients with psychotic depression and pre-existing psychosis. Nearly all patients in this study had poorly or only transiently responded to pretreatment with quetiapine. Special attention was paid to the tolerability of a direct treatment switch and the dose relationship between quetiapine and clozapine in PD.

## 2. Methods

Data from PD patients started on clozapine by the author, a movement disorder specialist certified in neurology and psychiatry and practicing in Vienna, between March 2003 and September 2023, were identified by retrospective review of patient records and copies of first clozapine prescriptions. For inclusion, patients had to have a diagnosis of PD according to the United Kingdom Brain Bank Criteria [15], a psychotic disorder and clozapine treatment for psychosis. Psychotic disorders included were either (1) PDPsy fulfilling DSM V-criteria in retrospect [16] with prominent visual hallucinations and/or delusions, (2) psychotic depression, or (3) pre-existing psychotic disorders. Patients with minor hallucinations only (illusions, passage, or presence hallucinations) [1], though some of them fulfilled NINDS-NIMH diagnostic criteria for PDPsy [2], were not deemed affected severely enough for clozapine treatment. To estimate the proportion of PD patients treated with clozapine for psychosis, these were compared with the total number of PD subjects first seen and followed up at least once by the author during the observation period which lasted until December 2023. PD patients who commenced on clozapine before March 2003 or by other physicians were excluded from further analysis.

Demographic data collected included pre-existing psychiatric disorders, age of PD onset, disease duration at the first occurrence of psychosis, spectrum of psychotic symptoms, and time course as well as triggering factors of psychosis. Previous antipsychotic treatments (especially treatment with and response to quetiapine), PD medication at the time of starting clozapine; mode of clozapine initiation; initial and maximum clozapine dose; dosing frequency; initial and long-term response including latency to first and maximum antipsychotic response, judged by the investigator as full (i.e,. complete resolution of psychotic symptoms), good (i.e., mild residual symptoms of PDPsy), moderate (i.e., relevant improvement, but marked residual symptoms of PDPsy), or poor (i.e., no clear-cut improvement of psychotic symptoms); adverse events and their management; and patient survival were reviewed. Levodopa equivalent doses of PD medication at the time of initiation of clozapine were calculated as described before [17]. For concomitant changes in the treatment of motor symptoms, antidepressants and cholinesterase inhibitors were documented.

Data were pseudoanonymized and the study was approved by the Ethics Committee of the City of Vienna (EK 23-189-VK). Due to the retrospective design and the lack of a control group, statistical analysis was limited to the calculation of mean values ± standard deviation for the age of PD onset, disease duration at the first occurrence of psychotic symptoms, time since the first occurrence of psychosis, duration of the current episode of psychosis, starting and maximum clozapine dose, duration of clozapine treatment and duration of PD, and age at the time of death in those deceased.

## 3. Results

A retrospective chart review identified 661 PD patients first seen and then followed between 2003 and 2023. Of them, 41 patients (17 female) were started on clozapine for psychosis by the investigator. Additional five patients with PDPsy were initiated on clozapine by other physicians, but data from those patients were only used to allow for an estimate of overall clozapine use for psychosis in PD (46 of 661 PD patients, i.e., approximately 7%).

The age at onset of PD in the 41 patients included was 59.1 ± 7.7 years (mean ± SD). Indications for clozapine were PDPsy (*n* = 36), in two of the cases with isolated visual hallucinations, in one with isolated delusions without hallucinations, and in 33 with both, hallucinations and delusions. Other indications included severe agitation associated with PDPsy (*n* = 1), severe hypersexuality with attempted rape in addition to PDPsy (*n* = 1), psychotic depression with suicidality (*n* = 2), and development of PD in a patient with schizoaffective psychosis (*n* = 1). Clinical data and details on antipsychotic treatment in 38 patients with PDPsy are summarized in Tables 1, 2, and 3. Illustrative case histories are presented in the Supporting Information (available here).

### 3.1. Patients With PDPsy

In those with PDPsy, the first psychotic symptoms were observed from 6 months to 29 years (9.8 ± 6.4 years) after the onset of motor symptoms. Latency from the first onset of psychosis to the commencement of clozapine ranged from 0.5 months to 18 years (3.0 ± 3.5 years). At the initiation of clozapine treatment, 8 patients suffered from acute PDPsy, 6 from long-standing PDPsy, 21 from an exacerbation, and 3 from a relapse of PDPsy. Duration of ongoing psychotic symptoms at the time of clozapine initiation ranged from 2 weeks to 3 years (6.5 ± 9.8 months). The most common trigger for psychosis at the time of clozapine initiation was dementia. Other important triggers included trauma, acute infections, and dopamine agonist treatment (see Table 1).

Of those with PDPsy, 37/38 had been pretreated with quetiapine at daily doses between 12.5 and 500 mg, in most cases with up to 150 mg per day. Response to quetiapine ranged from no response (*n* = 11), poor or moderate (*n* = 19), to transient (*n* = 8). Individual patients had been pretreated with other antipsychotics (see Table 1).

The majority of PDPsy patients (33/37) underwent an immediate overnight switch from quetiapine to clozapine, usually at a 3:1 or 4:1 ratio, as recommended previously [18]. Other cases were started at a low dose (6.25 or 12.5 mg) and uptitrated daily if they were inpatients or weekly in outpatients, in most cases at 12.5 mg steps. The starting dose in the PDPsy group was 37.7 ± 18.7 mg. A single patient who was switched from 150 mg quetiapine to 50 mg clozapine at bedtime reacted with troublesome sedation and stopped treatment. After months, a second treatment trial, starting with a reduction of quetiapine from 150 to 100 mg and an addition of 12.5 mg clozapine, again led to troublesome sedation and was aborted. All other 37 PDPsy patients tolerated the initial dose. The maximum clozapine dose ranged from 12.5 to 150 mg (72.9 ± 29.9 mg). The majority of patients showed an improvement in nighttime sleep after the first dose of clozapine. Latency to a meaningful response of psychosis ranged from 2 days to 6 months (see Table 1). Of the 37 PDPsy cases tolerating clozapine, 10 had a full, 23 had a good, 2 had a moderate, and 2 showed a poor clinical response to clozapine. Advancing dementia appeared to be the main reason for the lack of response.

Of the 37 patients who tolerated clozapine initially, only one (Case 2) opted to discontinue clozapine. In this case, trauma had led to severe psychosis with suicidal ideation. The patient responded well to clozapine and remained stable following clozapine withdrawal at 10 months. In two patients, clozapine had to be stopped for adverse events. In one patient (Case 23, see Supporting Information [available here]) the reason was agranulocytosis manifesting 8 weeks after starting clozapine. In another patient, thrombotic embolic pulmonary hypertension was diagnosed 8 months after initiating clozapine. Although the retrospective workup clarified that this condition must have originated before starting clozapine, it was withdrawn to minimize the risk of further thromboembolic events. Both patients were switched to quetiapine and suffered from several relapses of mild to moderate psychosis till death.

Other adverse events included a case of pulmonary embolism 1 month after clozapine initiation, potentially related to treatment and substantial weight gain (38% within 1 year) in the same subject. In addition to the subject with intolerable sedation (see above), 10 patients suffered from sedation which often improved after reducing the clozapine dose. Five complained of drooling and 2 complained of worsening orthostatic hypotension. In 20/38 PDPsy patients, no relevant side effects were documented. None of the patients showed worsening motor symptoms and several noted an improvement of tremor on clozapine.

By the end of the observation period, 25 patients with PDPsy had died, 9 were still followed, and 4 were lost to follow-up. Documented treatment duration in those initially tolerating clozapine ranged from 2 months to 12 years (30.6 ± 34.5 months). Last clozapine dose was 58.9 ± 33.5 mg. At this time point, 33/38 patients took a single clozapine dose at bedtime, 5 took a low dose in the late afternoon/early evening, and the larger dose was taken at bedtime. Long-term response in those taking clozapine for more than 6 months was full or good in 23/28 patients and moderate in 3/28 patients. Long-term response was poor in 2 PDPsy patients with progressive dementia who had shown a transient response in the beginning.

The 25 PDPsy patients who died during the observation period had a disease onset at 59.8 ± 7.6 years and experienced their first psychosis at the age of 70.7 ± 8.3 years. They started clozapine at 74.8 ± 7.3 years and were treated for 3.5 ± 3.2 years. Their age at death was 78.3 ± 6.6 years after a disease duration of 5–29 (18.9 ± 6.9) years. None of the deaths appeared to be related to clozapine treatment.

### 3.2. PD Patients With Other Psychotic Disorders

Two PD patients with psychotic depression with suicidality, in one case, evolving 4 years after the onset of motor symptoms, and in the other, before the onset of parkinsonism, switched to clozapine after prolonged treatment with quetiapine in one or with quetiapine and aripiprazole in the other case. One case showed a good and the other a moderate response to 50 mg clozapine. In both, suicidal ideation ceased with clozapine treatment. One patient with schizoaffective psychosis since early youth, progressive parkinsonism since the age of 47 years, and documented dopaminergic degeneration at age 57 showed an excellent motor and psychiatric response to a treatment change from risperidone and olanzapine to clozapine, followed by initiation of levodopa. For details, see case histories 39–41 in Supporting Information (available here).

## 4. Discussion

The high efficacy of clozapine in the treatment of PDPsy has been demonstrated in randomized, controlled [9, 10], and open-label studies [11, 13, 19–21]. Clozapine appears to be useful also in other psychotic disorders in PD including psychotic depression and pre-existing psychoses [22]. The present results further support the use of clozapine in psychotic disorders in PD, including patients with poor response to quetiapine. Although the proportion of PD patients treated with clozapine in this study (around 7%) is similar to another report from a specialized movement disorder center [13], clozapine was initiated often only after a prolonged course of psychosis and usually as second-line antipsychotic following quetiapine. The primary reasons for delaying clozapine were concerns about side effects and reluctance to undergo regular blood monitoring on the side of the patients and relatives, factors that lead to a severe underuse of clozapine [5, 14].

Adverse events observed in the present study were within the anticipated range. The risk of clozapine-induced agranulocytosis, possibly a toxic reaction related to the formation of nitrenium ions from clozapine metabolites in individuals with genetic susceptibility, is in the order of 0.5%–1%. Agranulocytosis typically evolves during the first 18 weeks on clozapine and few cases occur beyond 6 months of treatment [23]. Cases of neutropenia with later onset are often related to other causes such as viral infections. Late-onset neutropenia may not always warrant a termination of clozapine treatment [24]. A single patient in the study developed agranulocytosis 8 weeks after initiating clozapine. This event could be managed by discontinuing clozapine, treatment with filgrastim, a strategy recommended to shorten the neutropenic phase [23], and a direct antipsychotic switch back to quetiapine. Owing to mandatory weekly blood monitoring, the mortality of clozapine-induced agranulocytosis is substantially lower than the mortality of agranulocytosis related to other nonchemotherapy drugs (around 3% versus 7%–10% [23]). A single patient developed a pulmonary embolism 1 month after starting clozapine. Venous thromboembolism is a rare but serious adverse event associated with clozapine which may occur days to years after clozapine initiation, independent of clozapine dose [25].

The most common adverse effect, daytime sedation, often responded to a reduction of clozapine dose. Furthermore, single-dosing at bedtime can help to prevent this adverse event. Nevertheless, individual PD patients may not tolerate clozapine because of sedation, even when administered at bedtime and at low doses. Drooling and worsening of orthostatic hypotension can be troublesome side effects which may need specific management. Weight gain is one of the most common adverse effects of clozapine in psychiatric patients but is rarely observed in PD. In fact, a study comparing body weight in PD patients before and after starting clozapine found that PD patients continued to lose weight after treatment initiation [26]. Interestingly a single patient in the present study gained 38% body weight within a year on clozapine. Immobility after stopping an apomorphine pump may have contributed to weight gain in this case. Metabolic syndrome, dyslipidemia, insulin resistance, and new-onset diabetes are common side effects of clozapine in psychiatric populations. Although patients in the present study were not routinely screened for metabolic side effects, none developed diabetes mellitus. No adverse events related to high doses or rapid titration of clozapine such as seizures or myocarditis [27] were observed in the present study. Likewise, no case of hematological malignancy, a recently described long-term adverse event related to cumulative clozapine dose [28], was encountered.

All but one of the patients in the study were pretreated with quetiapine and many had received other antipsychotics. None of those pretreated were good long-term responders to quetiapine. Randomized, double-blind, placebo-controlled studies of quetiapine in PDPsy were negative [29–31], with the exception of a single small study showing an improvement in sleep and visual hallucinations by quetiapine given at bedtime [32]. Two randomized, but single-blinded studies comparing quetiapine and clozapine [33, 34] found that both drugs were effective in PDPsy, although clozapine was superior to quetiapine in one of the studies [34]. Despite limited evidence, quetiapine was labeled as possibly useful in an evidence-based MDS review [4] and is probably the antipsychotic most commonly used in PD outside the United States. Based on the author´s experience, quetiapine can effectively control hallucinations in many PD patients. Delusions tend to respond to quetiapine much rarer, an observation already made in previous studies [35]. We found that PDPsy rarely responded to an increase in quetiapine dose beyond 150 mg. Based on our own experience and expert opinion [36], we think that PDPsy patients should be switched to clozapine at the latest if they do not respond to a dose of 150 mg quetiapine. A direct overnight treatment switch from quetiapine to clozapine at a ratio of 3:1 or 4:1, as suggested previously [18], was tolerated by the vast majority of patients in the present study. This dose ratio can also be used when a switch back to quetiapine would become necessary, for example, in case of the development of severe neutropenia or agranulocytosis.

The inverse 5-HT2A receptor agonist pimavanserin, which has no dopamine receptor blocking activity, is the only approved drug for the treatment of PDPsy in the United States. Pimavanserin has a good safety profile and improved hallucinations, delusions, and nighttime sleep in a randomized placebo-controlled trial involving patients with mild to moderate PDPsy [37]. This was confirmed by a meta-analysis including unpublished prospective pimavanserin studies [38]. Although no data from prospective comparative trials are available, there is indirect evidence that clozapine may be superior to pimavanserin in terms of antipsychotic efficacy [39]. Moreover, uncontrolled studies show that PDPsy patients with the initial response to pimavanserin often need additional antipsychotic treatment later on [40] and that PDPsy refractory to pimavanserin may improve on clozapine [41].

Brexpiprazole is a partial dopamine D2 receptor agonist, recently approved in the United States for the treatment of agitation in Alzheimer's disease. Compared to aripiprazole, brexpiprazole has lower intrinsic activity at D2 receptors. A single case study found brexpiprazole helpful and tolerable in a 82-year-old PD patient with psychosis and delirium. However, this patient had been treated with unusually high doses of dopamine agonists [42]. By contrast, marked worsening of parkinsonism was observed in another single case following treatment with brexpiprazole [8]. Though low, the rate of extrapyramidal side effects in randomized controlled studies of brexpiprazole in Alzheimer's disease was still above placebo [7] and no data from prospective studies of brexpiprazole in PD are available.

Pretreatment with quetiapine, which in the end proved nonefficacious, led to the selection of a PD patient sample with severe psychosis, with all but two PDPsy patients suffering from delusions, and some of them showing severe agitation, violent behavior, and suicidality. Nevertheless, almost 90% of the PDPsy patients tolerating clozapine showed full or good response and almost 80% of the patients showed good long-term response of psychosis. However, the average dose of clozapine in the present study (72.9 mg at maximum) needed for effective control of psychosis was substantially higher than the doses used in randomized studies. Insomnia, vivid dreams, and nighttime agitation often responded to the first dose of clozapine. Hallucinations usually improved within a few days to 2 weeks. By contrast, delusions, particularly those unrelated to hallucinations, sometimes only gradually improved and occasionally only resolved after months or even years of clozapine treatment (see, for example, Case 16). This parallels observations in schizophrenia, where the latency to the response of delusions to antipsychotics is longer than the time to the improvement of hallucinations [43] and the process of amelioration of delusions is often gradual with abnormal thoughts first becoming less pressing and delusions sometimes only resolving after months of antipsychotic treatment [44].

According to the motivational salience hypothesis [45], dopamine mediates the conversion of neural representations of external stimuli from neutral information into an attractive or aversive entity, that is, it confers salience to perceptions and ideas. Psychosis in schizophrenia results from a hyperdopaminergic state lending increased, aberrant salience to experiences in the environment and internal representations. Antipsychotics, by blocking dopamine D2 receptors, dampen the abnormal salience of these experiences [44]. In a patient with delusions, antipsychotics do not primarily change the content of the delusional thoughts or ideas but first reduce the degree to which the symptoms occupy the mind and distress the patient, meaning that the abnormal thoughts may be still there but not important any more. Only later, over weeks, the fundamental content of the delusions may be deconstructed and vanish completely [44]. These mechanisms may be relevant to PDPsy as well, in particular for delusions not directly related to hallucinations.

Suicide is generally held as a rare event in PD. However, studies have shown an increased rate of suicide in PD patients following deep brain stimulation surgery. A recent meta-analysis found a higher risk of suicidal behavior in PD as compared to controls [46]. In line with these data, both patients with psychotic depression and other patients with PDPsy included in the present study were suffering from suicidality. Clozapine is among the few psychopharmaceutical options with a clear role in suicide prevention in psychiatric populations [47]. Clozapine's beneficial effect on suicidality may also be relevant to PD patients with psychosis.

Dementia appeared to be the main triggering factor for psychosis in the present study. Also, poor response to clozapine, observed in a small minority of patients, most likely was related to progressive dementia. Of particular interest in this regard was a single patient with early hallucinations on dopamine agonist treatment and the development of dementia and severe PDPsy (responding to clozapine) only 3 years after the onset of PD at the age of 63 years. Genetic testing within another research programme showed a heterozygote GBA mutation (E326K), which may have contributed to the aggressive course of his parkinsonism and early development of PDPsy. The vast majority of PDPsy patients in this series received cholinesterase inhibitors. A recent meta-analysis confirmed that cholinesterase inhibitors improve hallucinations and delusions overall. However, unfortunately, their effect size on psychotic symptoms in PD is low [48].

This study has several limitations. It was a retrospective, open-label, single-observer study with a restricted number of patients. Due to the retrospective nature of the study and the complexity of psychotic disorders in PD, initiation of clozapine was accompanied by changes in medication for motor symptoms, treatment of other triggering factors, and initiation of cholinesterase inhibitor treatment in some of the patients. Natural fluctuations in the severity of psychosis and placebo response have to be considered as contributors to the outcome. Consequently, the antipsychotic response in this study cannot be fully attributed to clozapine. However, the majority of patients had a prolonged latency from the first psychotic symptoms to the initiation of clozapine, which in many cases had led to a reduction of adjuvant PD medication with a high potential to induce psychosis before starting clozapine. To exemplify, only one of the 38 patients with PDPsy was on an anticholinergic at the time of starting clozapine and only 10/38 were still on an oral dopamine agonist.

Since patients were followed in a routine setting, no psychometric scale to assess psychotic symptoms and no standardized instruments to document adverse events were used. Furthermore, clinic or practice visits did not follow a fixed schedule. Due to these methodological limitations, the lack of an a priory defined hypothesis, and the lack of a control group, no formal statistical testing of outcomes was performed. However, patients and caregivers were routinely questioned for the presence of hallucinations and delusions in the time period before each clinical follow-up. Improvement or worsening of psychotic features and adverse events were meticulously recorded.

The strengths of the present study are the standardized documentation and treatment approach provided by a single observer, the detailed description of psychopathology and its response to clozapine, the long clinical follow-up with observed clozapine treatment times of up to 12 years, and the inclusion of patients with severe psychotic disorders poorly responsive to quetiapine, and often requiring higher doses of clozapine than in randomized studies.

Our observations lend further support to the usefulness of clozapine in the treatment of PDPsy and other psychotic disorders in PD including psychotic depression and pre-existing psychoses. We found clozapine to be effective in patients with psychoses poorly or only transiently responding to quetiapine. A treatment switch to clozapine should be considered if other measures against PDPsy are not effective. Hallucinations may respond earlier than delusions. Adverse events including agranulocytosis are manageable in the majority of patients.

## Figures and Tables

**Table 1:** Clinical characteristics of 38 patients with PD psychosis before starting clozapine.

Case no.	Sex	Age of PD onset	Neuropsychiatric disorders before PD onset	PD duration at first-time PDPsy (yr)	Time since first PDPsy (mo)	Duration of present PDPsy before CLO (mo)	Course of present PDPsy	Psychotic symptoms present PDPsy	Triggering factors for present PDPsy	PD medication (daily dose in mg)	LEDD (mg)
1	m	43		8	4	4	Ongoing	VH, AH, D (persecution, poisoning)	Dementia	Dopa 100, CR 100, LCE 550	940
2	f	61		11	0,5	0,5	Acute onset	VH, D (persecution)	Sternum fracture	Dopa 450, RAS 1, ROP 16, AMA 300	1170
3	m	69		6	60	6	Exacerbation	VH, D (persecution)	Dementia	Dopa 600	600
4	m	53		15	36	10	Exacerbation	VH, D (persecution)	Insomnia, nocturnal confusion	Dopa 600 (pump), CR 100	675
5	f	62		7	72	6	Exacerbation	VH, AH, D (persecution, poisoning)	Dementia	Dopa 1000, CR 100	1075
6	f	49		5	216	1	Exacerbation	VH, AH, D (jealousy)	Dementia	Dopa 175, OPI 50, RAS 1, AMA 200	563
7	m	50	Depression	15	36	0,5	Exacerbation	VH, D (persecution, jealousy)	Apomorphine pump	APO 204 (pump), dopa 250, CR 100, RAS 1	2465
8	f	53	Personality disorder	6	120	1	Exacerbation	VH, D (persecution, jealousy)	Personality disorder	CR 200, LCE 900, RAS 1, AMA 100	1547
9	f	71	Depression	8	24	2	Exacerbation	VH, D (persecution, harm)	Dementia	Dopa 1350, CR 200, RAS 1	1600
10	m	67		9	1	1	Acute onset	VH, D (persecution, harm)	Dementia	LCE 686 (pump), CR 100	761
11	m	60	Depression	0,5	31	2	Relapse	VH, AH, OH, TH, D (persecution)	Dementia, GBA mutation	Dopa 750, AMA 200	950
12	f	50	Depression	16	15	1	Exacerbation	VH, AH, D (persecution, harm, poisoning)	Apomorphine pump	APO 56 (pump), dopa 500	1060
13	m	63		1	48	9	Exacerbation	VH, AH, D (persecution)	Dementia	Dopa 450	450
14	m	59		0,5	28	0,5	Exacerbation	VH, AH, D (persecution)	Dementia	Dopa 500, CR 100, RAS 1	675
15	f	65	Depression	10	36	36	Ongoing	VH, AH, D (persecution)	Dementia	Dopa 450	450
16	m	52		12	15	15	Ongoing	D (jealousy, technical)	None	Dopa 800, CR 200, RAS 1, ROP 3, AMA 200	1310
17	f	54		6	1	1	Acute onset	VH, AH, D (persecution, harm)	DDS, PPX	Dopa 500, CR 200, LCE 1200, PPX 2	2611
18	f	57	Depression, anxiety	8	1	1	Acute onset	VH, D (persecution)	Acute spondylodiscitis	APO 150 (pump), dopa 600, CR 100, RAS 1	2275
19	f	70		13	6	0,5	Exacerbation	VH	Dementia, PPX	Dopa 425, CR 200, PPX 2, AMA 200	975
20	m	45		22	36	12	Exacerbation	VH, D (persecution)	Dementia	LCE 750, dopa 100, AMA 300	1431
21	m	57		7	72	1	Exacerbation	VH, AH, D (persecution, poisoning)	Dementia	LCE 1546 (pump)	2056
22	f	50		29	36	24	Exacerbation	VH, D (persecution)	Dementia	Dopa 50, LCE 300, RAS 1	566
23	m	53		23	2	2	Acute onset	VH, AH, D (persecution)	Dementia, PPX	Dopa 400, CR 100, LCE 800. SEL 5, PPX 0.75	1796
24	m	67		14	120	0,5	Relapse	VH, TH, D (persecution)	Dementia, mild TBI	Dopa 50, CR 200, LCE 350, RAS 1	782
25	m	65		4	24	3	Exacerbation	VH, D (persecution)	Dementia	Dopa 250, LCE 600, AMA 100	1231
26	m	55	Depression, TBI	4	48	0,5	Exacerbation	VH, D (persecution)	Dementia	Dopa 900 (pump), AMA 100	1000
27	f	59		9	2	2	Acute onset	VH, AH, D (persecution)	Spine fracture, UTI, PPX	Dopa 750, RAS 1, PPX 4.5, AMA 400	1700
28	f	62		5	24	24	Ongoing	VH, D (jealousy, harm)	Dementia	Dopa 1250, CR 200, OPI 50, RAS 1, AMA 100	2225
29	m	45		4	48	2	Exacerbation	VH, AH, D (persecution, religious)	Procyclidine, CAB	Dopa 500, CR 100, CAB 4, procyclidin 5	839
30	m	68		12	13	1	Exacerbation	VH, AH, TH, D (persecution, harm, parasite)	Dementia	Dopa 750, CR 200, RAS 1, AMA 100	1100
31	m	69		8	18	1	Exacerbation	VH, D (persecution, parasite)	Dementia	Dopa 600, RAS 1	700
32	m	70		14	36	12	Relapse	VH, D (jealousy)	Dementia	Dopa 250	250
33	f	64		17	1	1	Acute onset	VH, D (persecution)	Dementia, PPX	LCE 600, PPX 1	898
34	m	73		1	12	1	Exacerbation	VH, D (persecution, harm)	Dementia	LCE 500	665
35	m	57		15	60	0,5	Exacerbation	VH, D (persecution)	Dementia, PPX	Dopa 300, CR 200, LCE 600, PPX 4.5, AMA 300	2097
36	f	62		9	24	24	Ongoing	VH	Dementia	Dopa 25, LCE 500, RAS 1	798
37	f	58		3	36	36	Ongoing	VH, D (persecution)	Dementia	Dopa 1350, CR 200	1500
38	f	58		14	1	1	Acute onset	VH, D (persecution, jealousy)	Dementia, ROP	Dopa 200, LCE 800, ROP 2	1370

*Note:* AD, auditory hallucinations; CR, controlled release levodopa; GBA, glucocerebrosidase B.

Abbreviations: AMA, amantadine; AP, antipsychotic; APO, apomorphine; CLO, clozapine; D, delusions; DDS, dopamine dysregulation syndrome; f, female; LCE, levodopa, carbidopa, entacapone; LEED, levodopa equivalent dose; m, male; mo, months; OH, olfactory hallucinations; OPI, opicapone; PD, Parkinson's disease; PDPsy, Parkinson's disease psychosis; PPX, pramipexole; RAS, rasagiline; ROP, ropinirole; TBI, traumatic brain injury; TH, tactile hallucinations; UTI, urinary tract infection; VH, visual hallucinations; yr, years.

**Table 2:** Antipsychotic treatment before starting clozapine in 38 patients with PD psychosis.

Case no.	Previous AP pretreatment (max. daily dose)	Response to QUE	Switch from QUE to CLO
1	QUE 425	Poor	425–100 mg
2	None	Poor	No
3	QUE 150	No	150–50 mg
4	QUE 275	Poor	150–37.5 mg
5	QUE 100	No	100–25 mg
6	QUE 150	Moderate	150–50 mg
7	QUE 150	Moderate	150–50 mg
8	QUE 150	Poor	150–50 mg
9	QUE 150	Poor	150–50 mg
10	QUE 150	Poor	150–50 mg
11	QUE 25	No	25–12.5 mg
12	QUE 500	No	200–50 mg
13	QUE 25	Worsening	No
14	QUE 150	Poor	150–50 mg
15	QUE 200	Transient	200–50 mg
16	QUE 100	No	No
17	TIA 200, QUE 25	No	25–25 mg
18	QUE 100	No	100–25 mg
19	OLA 2.5, QUE 150,	No	150–50 mg
20	HAL 10, RIS 1, QUE 150	Transient	150–50 mg
21	QUE 200	Moderate	200–50 mg
22	QUE 200	Moderate	100–25 mg
23	2.5 OLA, 80 PRO, QUE 150	Moderate	Within days
24	100 QUE, HAL 5	Transient	100–25 mg
25	QUE 100	Poor	100–37.5 mg
26	QUE 100	Transient	100–25 mg
27	QUE 100	Poor, moderate later on	100–25 mg
28	QUE 200	Transient	200–50 mg
29	QUE 150	No	150–50 mg
30	QUE 150	Moderate	150–50 mg
31	RIS 1.5, QUE 150	Transient	150–50 mg
32	QUE 250	Moderate	250–50 mg
33	QUE 25, RIS 0.5	Poor	25 QUE and 0.5 RIS to 25 mg
34	QUE 50	No	50–12.5 mg
35	QUE 275	Transient	275–50 mg
36	QUE 150	Poor	150–50 mg
37	QUE 112,5	Transient	112.5–6.25 mg
38	QUE 100	Poor	50–25 mg

Abbreviations: AP, antipsychotic; HAL, haloperidol; OLA, olanzapine; PD, Parkinson's disease; PRO, prothipendyl; QUE, quetiapine; RIS, risperidone; TIA, tiapride.

**Table 3:** Response to clozapine in 38 patients with PD psychosis.

Case no.	CLO starting dose (mg)	CLO max daily dose (mg)	Last observed daily CLO dose (mg)	Time of CLO intake	Duration of CLO treatment (mo)	Reason for stopping CLO	Patient status at the time of study	Age at death (yr)	PD duration at death (yr)	Response to CLO	Latency to first response to CLO	Long-term CLO response	Adverse events on CLO
1	100	125	125	Nighttime	12		CLO ongoing			Full	4 weeks	Full	None
2	12.5	62.5	25	Nighttime	10	Patient preference	Psychosis resolved			Good	1 week	Good	Sedation
3	50	62.5	56.25	6 p.m. and nighttime	20		CLO ongoing			Good	2 weeks	Good	Sedation
4	37.5	50	50	Nighttime	4		CLO ongoing			Full	Days	NA	None
5	25	25	25	Nighttime	4		CLO ongoing			Full	2 weeks	NA	None
6	50	50	12,5	Nighttime	0	AE	Recurrent psychosis			NA	NA	NA	Sedation
7	50	100	75	Nighttime	8		CLO ongoing			Full	2 weeks	Good	None
8	50	50	12.5	Nighttime	5		CLO ongoing			Good	Gradual over 3 months	NA	Sedation
9	50	62.5	62.5	Nighttime	6		Lost to follow-up			Delayed good	2 weeks	NA	None
10	50	68.75	43.75	5 p.m. and nighttime	25		CLO ongoing			Full	1 week	Full	None
11	12.5	50	50	Nighttime	18		Deceased	64	5	Good	2 weeks	Good	Drooling
12	50	150	150	Nighttime	8		Lost to follow-up			Good	1 week	Poor	None
13	12.5	12.5	12.5	Nighttime	4		Lost to follow-up			Good	2 weeks	NA	None
14	50	75	50	6 p.m. and nighttime	31		Lost to follow-up			Good	Days	Good	None
15	50	100	100	Nighttime	24		Deceased	81	17	Poor	NA	Poor	None
16	6.25	75	25	Nighttime	140		Deceased	77	25	Delayed good	Months	Full	Sedation, drooling
17	25	100	50	Nighttime	53		Deceased	64	10	Full	Gradual over 1 month	Good	None
18	25	125	125	6 p.m. and nighttime	58		Deceased	80	23	Good	1 week	Good	PE, weight gain
19	50	75	75	Nighttime	26		Deceased	85	15	Good	2 weeks	Moderate	None
20	50	75	62.5	Nighttime	13		Deceased	71	25	Good	Days on 75 mg	Good	Drooling, worsened OH
21	50	100	100	Nighttime	6		Deceased	70	13	Good	1 week	NA	Sedation
22	25	50	50	6 p.m. and nighttime	52		Deceased	82	32	Good	1 week	Good	None
23	12.5	50	50	Nighttime	2	AE	Deceased	81	28	Good	1 week	NA	Agranulocytosis
24	25	100	100	Nighttime	8		Deceased	85	18	Good	Days	Good	None
25	37.5	50	50	Nighttime	47		Deceased	76	10	Full	1 week	Good	None
26	25	75	37.5	Nighttime	94		Deceased	77	22	Full	Days	Good	Sedation
27	25	25	12.5	Nighttime	8	AE	Deceased	77	18	Full	Days	Good	TE-PH, likely unrelated
28	50	100	100	Nighttime	45		Deceased	79	16	Transient poor	NA	None	Sedation
29	50	50	25	Nighttime	145		Deceased	74	29	Good	2 weeks	Good	None
30	50	100	75	Nighttime	59		Deceased	86	17	Good	Gradual over 3 months	Good	Sedation, drooling
31	50	100	100	Nighttime	47		Deceased	82	13	Good	1 week	Good	Drooling
32	50	100	50	Nighttime	6		Deceased	88	18	Moderate	1 week	NA	Sedation
33	25	37.5	25	Nighttime	36		Deceased	84	20	Good	1 week	Full	Worsened OH
34	12.5	50	50	Nighttime	34		Deceased	77	10	Good	1 week	Full	None
35	50	50	50	Nighttime	44		Deceased	81	24	Full	Gradual over 4 weeks	Good	None
36	50	75	75	Nighttime	11		Deceased	73	11	Good	6 months	Moderate	Sedation
37	6.25	100	50	Nighttime	107		Deceased	83	25	Moderate	4 weeks	Moderate	None
38	25	62.5	50	Nighttime	19		Deceased	84	26	Good	1 week	Good	None

Abbreviations: AE, adverse events; CLO, clozapine; max, maximum; mo, months; NA, not applicable; OH, orthostatic hypotension; PD, Parkinson's disease; PDPsy, Parkinson's disease psychosis; PE, pulmonary embolism; TE-PH, thrombotic embolic pulmonary hypertension; yr, years.

## Data Availability

All relevant data are included in the manuscript and supporting information.

## Supporting Information

The supporting file provides a detailed description of the course and treatment of psychosis in six illustrative patients with Parkinson psychosis (Cases 8, 11, 16, 20, 23, and 25), two patients with psychotic depression (Cases 39 and 40), and a patient with PD and pre-existing schizoaffective psychosis (Case 41 of the manuscript).
