# Supplementary material for: Clozapine for Quetiapine-Refractory Psychosis in Parkinson's Disease: A Long-Term Single-Center Retrospective Study
Source: Parkinsons Dis. 2025 Mar 10;2025:1068722. doi: 10.1155/padi/1068722 (PMC11991777; doi:10.1155/padi/1068722)
Supplement: Supporting Information — Additional supporting information can be found online in the Supporting Information section. [file 1068722.f1.pdf]

Clozapine for quetiapine-refractory psychosis in Parkinson's disease: A long-term retrospective study

Walter Pirker

Supplementary material: Illustrative case histories

Case 8

At the age of 52 a female patient with preexisting personality disorder presented with parkinsonism. She responded well to levodopa and developed motor complications. Eight years into the disease she appeared increasingly jealous. She displayed aggressive outbursts and in public accused her spouse of extramarital relationships. Soon she felt observed by people that were standing at the opposite building's windows. Shielding the windows and moving to another flat did not improve the situation. After ten years of delusional jealousy of fluctuating severity the patient experienced a severe exacerbation of her psychosis with formed visual hallucinations of people, auditory hallucinations of voices, vivid delusional perceptions and worsening of her delusional jealousy. Her fear escalated and following a heated discussion with her daughter she intended to jump from her flat‘s balcony. After a short evaluation at a psychiatric department she was treated with lorazepam and admitted to a neurological ward where she appeared mildly confused, restless and mistrustful towards her relatives. She showed hastened, somewhat incoherent thinking, frequent illusions and auditory hallucinations, though not in the form of voices. Stopping rasagiline and amantadine and a fast titration of quetiapine to 150mg did not improve her psychosis. A direct switch to 50mg clozapine was well tolerated and led to rapid resolution of illusions and hallucinations. Delusions of jealousy and persecution markedly improved over the following months. Half a year after starting clozapine, the patient showed normal cognition, no suicidal ideation and had a stable marital relationship.

Case 11

This male patient fist noted intermittent cramping and tremor of his left hand at age 60. His father had died aged 86 years after four years of progressive parkinsonism. Pramipexole led

to a moderate improvement of motor symptoms. However, within two months the patient started to experience his environment as aggressive and felt disturbed by music. He noted low-pitched voices and clicking in plug-sockets and, from time to time, an irritating sweet smell. At times he felt observed as an object of an experiment. At other times he felt involved in the activity of a drug syndicate. Friends noted that the patient switched between a severe delusional state and periods with preserved insight into the pathological character of these perceptions and thoughts. A changeover from pramipexole to levodopa and short-time treatment with 12.5mg quetiapine at bedtime led to a rapid resolution of psychosis and an improvement in motor symptoms. However, he continued to suffer from disturbing rest tremor. Two years later amantadine was added for dyskinesias. Two and a half years after starting levodopa, whilst still on amantadine, the patient suffered a severe relapse of psychosis. He described shadow images of people, sometimes resembling his father. From time to time the shadow people talked to each other, at other times the images touched him, for example by placing their hand on his shoulder. When working on his computer the patient often noted objects in his peripheral visual fields. Following the moving-in of a new neighbour he often overheard voices talking through the wall. He became convinced that his new neighbour intended to bully him out of his apartment. He considered calling in the police but then suddenly realized the delusional nature of this idea and attended a neurological follow up visit. Since short-time treatment with 25mg quetiapine before sleep had no effect, he was admitted and switched to clozapine. Amantadine was stopped. Within two weeks, a gradual increase of clozapine dose from 12.5 to 50mg led to a marked improvement of psychosis and of tremor. Since mild Parkinson‘s dementia was evident at that time, he was started on rivastigmine. Despite good control of the psychosis, cognition continued to rapidly decline. The patient developed non-fluent aphasia and died 18 months after starting clozapine. Exome sequencing, performed within a research programme, revealed a heterozygote GBA gene mutation (E326K).

Case 16

At the age of 52, a skilled technician developed parkinsonism. He responded very well to levodopa and dopamine agonist treatment. Twelve years after onset, during a group travel he observed for the first time that fellow travellers were able to communicate with each

other using an invisible microphone. He also sensed that people's hand movements could induce galvanic currents allowing for remote control of this communication. Following this experience he started to observe the same behaviour in his wife. During the subsequent months the delusional perception of his wife communicating with other men via an invisible device and using subtle changes in upper limb positions worsened leading to feelings of jealousy and severe emotional distress in both partners. Withdrawal of ropinirole led to mild motor worsening but didn't improve psychosis. Regular psychotherapy discussing the delusional character of the perceptions and treatment with quetiapine brought no sustained improvement. A dose increase of quetiapine to 100mg at night-time led to sedation and the patient stopped treatment. One year after onset of the psychosis, the patient's wife appeared to communicate throughout the whole day using specific mouth and hand positions. Clozapine was started at a very low dose and gradually increased. 75mg at night-time led to some sedation which abated after reducing the dose to 50mg. The delusional perception persisted for about four more years but tended to occur less frequently over time and, according to the patient, became irrelevant for him after some months of treatment with clozapine. Motor symptoms slowly worsened. The patient developed mild dementia at age 76 and died from aspiration pneumonia one year later. He was still on clozapine at the time of death and had never experienced hallucinations.

Case 20

This male patient with a family history of Parkinson's disease developed parkinsonism at age 45 and responded well to levodopa. Approximately 22 years after onset the patient showed the first signs of mild dementia and had a first psychotic episode with vivid hallucinations of people and animals. This was treated with risperidone and an anticholinergic. Two years later the patient was admitted to a traumatology ward after a fall and treated with intravenous haloperidol for severe night-time agitation owing to persecutory delusions. During subsequent treatment in a psychiatric hospital he improved on quetiapine. However, hallucinations of people and animals soon recurred. The patient started to feel observed and displayed hypersexual behaviour. Stopping selegiline, reducing pergolide, adding donepezil and increasing the dose of quetiapine led to a marked improvement in psychosis. However, hypersexuality only improved following withdrawal of pergolide but this led to a marked

worsening of his motor symptoms. The addition of a COMT inhibitor triggered a recurrence of hypersexuality which resulted in the attempted rape of his wife. A transition from 150mg quetiapine to 50mg clozapine, followed by an increase of clozapine to 75mg at night-time rapidly led to a good and prolonged control of hypersexuality. Side effects included severe orthostatic hypotension and drooling. Further worsening of cognition and mobility led to his death 25 years after onset of parkinsonism.

Case 23

This man presented with an akinetic crisis at age 75. He had a 22 year history of PD with mild cognitive decline over the last 10 years. He responded well to a moderate increase in levodopa and addition of a COMT inhibitor. However, within a few weeks the patient developed a severe psychosis with visual hallucinations of people, sometimes telling him to be careful with water, sometimes of men dressed in black, threatening to attack the patient. He described intermittent episodes when he felt controlled by foreigners or surrounded by enviers. He remarked that his brain tended to switch on and off suddenly. He was hospitalized after he fled his home due to persecutory delusions. Stopping low-dose pramipexole and selegiline and adding quetiapine up to 150mg at night-time led to a moderate improvement in psychosis but intermittent delusions of poisoning as well as nocturnal agitation persisted. A switch from quetiapine to 50mg clozapine at night-time led to better sleep, a complete resolution of psychotic symptoms within days and improvement in his tremor.

Eight weeks after starting clozapine the weekly complete blood count showed 1.700 leukocytes/mcl and an absolute neutrophil count of 37/mcl (baseline values before starting clozapine 5.400 and 4.100/mcl, respectively). The patient reported diarrhea and mild headache for four days but showed no signs of systemic infection. He was admitted to hospital, isolated and received short-time prophylactic antibiotic treatment. Following a direct switch from 50mg clozapine to 150mg quetiapine and treatment with filgrastim, a recombinant granulocyte colony stimulating factor, neutrophil count increased to 5.070/mcl on day 3 and 29.770/mcl on day 5 but returned to 5.830/mcl on day 8 and the patient could be discharged. On quetiapine, he suffered from recurrent psychosis over the following years and died at age 81.

Case 25

This male patient developed mild hypophonic dysarthria and a slow gait at the age of 65 and was diagnosed with mixed type Parkinson's disease one year later. Low dose levodopa and amantadine (200mg daily dose, each) brought little improvement and soon the patient noted occasional visual illusions in the form of mild make-believe movements of stationary objects. A gradual increase of levodopa dose to 600mg and addition of rasagiline led to clear-cut improvement of motor symptoms. However, four years after onset the patient showed signs of mild dementia with intermittent confusion. While on levodopa, rasagiline and amantadine illusions began to occur regularly and the patient first noted formed visual hallucinations of various people. Stopping rasagiline, adding rivastigmine and low dose quetiapine kept illusions and visual hallucinations unintrusive with retained insight for about two years. After adding entacapone for severe motor fluctuations six years after onset of PD, the patient suffered an exacerbation of his psychosis with daily vivid hallucinations of zombies which seemed to live as lodgers in the patients flat and misplaced his objects. Reducing amantadine and an increase of quetiapine to 100mg at night brough no relief. The patient noted frequent nightmares with a voice telling him he should not get up, otherwise he would die. Especially in the late afternoon hours he often observed little reptiles and up to 15 people wrapped in plastic in his flat which, according to him, belonged to a subterranean subculture. In his sons' bed the patient sometimes saw several zombies at the same time. Once the patient awoke during the night with the sensation of a gecko sitting on top of his eye. Insight into the irreal character of these frightening perceptions waxed and waned. There were no delusions. A switch from quetiapine to 37.5mg clozapine at night led to marked improvement of hallucinations within one week without side effects. Mild unformed hallucinations (shadows accompanying the patient) persisted. Over the following years the patient intermittently noted mild hallucinations (illusions, presence hallucinations) and short-time worsening of his psychosis with the recurrence of formed visual hallucinations of people and feelings of persecution. The patient died ten years after onset of PD, bedridden and severely demented.

Case 39

This male patient suffered from recurrent depression since age 41. At 57 he developed a shuffling gait and a reduced arm swing on the right. Two years later he was diagnosed with

PD. Given his comorbid depression, he was treated with ropinirole which was tolerated apart from occasional feelings of another person in the room or behind his back. At the age of 61 the patient noted recurring visual illusions, e.g. perceiving a water hydrant as a little boy or a coat hook as an adult. He developed depression, progressive insomnia and panic attacks at night. In addition there were severe delusions of poverty congruent with depression and frequent suicidal thoughts. Following poor response to antidepressants and quetiapine up to 250mg he was switched to 50mg clozapine which was well tolerated and led to a marked improvement of the psychosis. Clozapine treatment also allowed for a substantial increase in levodopa which led to a marked improvement of motor symptoms. Clozapine was continued for approximately 7 years (dose at last observation 25mg). The patient continued to suffer from mild to moderate depression and developed moderate dementia but stayed free of hallucinations and delusions.

Case 40

At the age of 61 this patient developed severe depression with suicidal ideation and delusions of poverty. Following treatment trials with several antidepressants and antipsychotics over months asymmetric parkinsonism became evident. Dopamine transporter imaging revealed asymmetric striatal nerve terminal loss. The patient was started on 300mg levodopa daily, initially without improvement. A year after onset of depression he was switched from 200mg quetiapine and 15mg aripiprazole to 50mg clozapine. This was tolerated without side effects and led to a minor improvement of psychotic depression and motor symptoms within 4 weeks. Following a gradual increase of levodopa to 600mg/day and 6 months after starting clozapine motor symptoms were moderately improved. The patient still appeared moderately depressed but delusions of poverty and suicidality had subsided.

Case 41

This patient suffered from schizoaffective psychosis since early youth with severe bouts of dysphoric mania. He was treated with a range of antipsychotics including haloperidol decanoate injections. At the age of 47, he developed slowly progressive parkinsonism with

rest tremor. This improved but did not completely resolve after switching from haloperidol to combinations of low dose risperidone (1mg/day) with quetiapine and, later with olanzapine. Attempts to withdraw risperidone led to dysphoric mood states and bursts of aggression. Dopamine transporter imaging, performed at age 57 for worsening of parkinsonism, revealed asymmetric striatal binding loss indicative of PD. A gradual treatment change from 1mg risperidone and 22.5mg olanzapine to 50mg clozapine over 2 months, followed by levodopa initiation (300mg/day) was well tolerated and led to a marked improvement of parkinsonism. At a dose of 25mg clozapine in the morning and 87.5mg at bedtime affective and psychotic symptoms have been relatively well controlled 4 years since initiating clozapine.
